# The Validity of a Smartphone-Based Method for Acquiring 3D Images of the Face

**DOI:** 10.3390/jcm13216362

**Published:** 2024-10-24

**Authors:** Alexandra K. Papadopoulou, Francesca Di Santo, Gregory S. Antonarakis, Luis Huanca Ghislanzoni

**Affiliations:** 1 Division of Orthodontics, University Clinics of Dental Medicine, Faculty of Medicine, University of Geneva, 1 Rue Michel-Servet, 1211 Geneva, Switzerland; francesca.disanto@outlook.com (F.D.S.); gregory.antonarakis@unige.ch (G.S.A.); luis.huanca@unige.ch (L.H.G.); 2 Discipline of Orthodontics, Sydney Dental School, Faculty of Medicine and Health, The University of Sydney, Sydney, NSW 2010, Australia

**Keywords:** face scanning, 3D face, smartphone apps, smartphone face scan, accuracy, anthropometry

## Abstract

**Objectives.** To evaluate the accuracy and reproducibility of measurements obtained using the Bellus3D Face Application on a mobile smartphone by comparing them to direct measurements on pre-marked and blank face scans. **Materials and Methods.** Twenty-five healthy young adults (six males and nineteen females; age range 20–30 years) were included in this prospective cross-sectional study, with the only exclusion criterion being the presence of significant facial hair interfering with the placement and visualization of landmarks. Image acquisitions were performed using an iPhone XR with the Bellus3D FaceApp face scanning application, an iOS application for smartphones. Ten single midfacial and five paired bilateral landmarks were defined and marked. Two face scans were performed on each patient, both on blank and marked faces, and distances were measured directly with calipers and digitally. **Results.** The random error values were 1.0 mm and 0.4 mm for the manual point placement and measurements and virtual point placement on blank faces, respectively. The two methods used (the direct method and acquisition on faces with landmarks) demonstrate relatively similar reliability (ICC > 0.8); however, a paired *t*-test showed that the differences between several measurements were statistically significant (*p* < 0.05). Regardless of the method used, there was a systematic error for various values that included the nose and mouth (*p* < 0.05). The measurements demonstrating the most significant differences between the methods were those that included the tip of the nose, with the mean differences being −4.4–3.3 mm. The measurements of the distances that estimate face “depth” showed the greatest consistency irrespective of the tested method (*p* > 0.05 and ICC > 0.8). **Conclusions.** The use of the Bellus3D FaceApp is precise and reproducible for certain areas of the face, but digital reconstruction errors prohibit, for the time being, the use of this technology in everyday clinical practice. The noted discrepancies were consistent and more prevalent for specific areas such as the tip of the nose. Further investigations are required to determine other sources of error and for other smartphone-based applications released for 3D face image acquisitions.

## 1. Introduction

Numerical measurements of the human face have been a source of interest in various fields of science, medicine, and dentistry as part of the diagnosis and digital workflow for treatment planning [1,2]. Facial anthropometry enables the diagnosis of different syndromes, the estimation of normal and abnormal growth, and the planning and evaluation of conventional orthodontic interventions either alone or in combination with orthognathic surgery [3]. Thus, the development of systems that enable precise anthropometric measurements is growing exponentially, not only in capturing and assessing static images but also the dynamic conditions of the face, such as movement, while performing various facial expressions, such as talking and smiling [4,5].

Several techniques have been applied for 3D reconstruction of the face, including methods that use radiation exposure [computed tomography (CT) and cone-beam computed tomography (CBCT)], as well as ultrasound, infrared, laser scanning, magnetic resonance imaging, and stereophotogrammetry [3,4,6,7,8,9]. Stereophotogrammetry appears to be a promising approach and the method of choice for face capturing and measurements [3,10,11] due its nature not including radiation exposure and its accuracy being sometimes even better than direct measurements [6,7,12,13,14,15]. More specifically, de Menezes et al., 2010 [3] investigated the accuracy and reproducibility of the Vectra 3D imaging system, which consists of two pods, three cameras (two black-and-white and one color) and a projector in each pod. The sample consisted of 10 healthy subjects on which 50 facial soft tissue landmarks were marked with a black liquid eyeliner, and 16 linear measurements were performed digitally after image acquisition. It was found that 3D stereophotogrammetry using the Vectra 3D imaging system provides a quick, precise, and repeatable assessment of facial landmarks, with random errors being less than 1 mm. Weinberg et al., 2006 [13] investigated the accuracy and precision of measurements performed by two different digital 3D photogrammetry systems (Genex and 3dMD) and direct anthropometric measurements with calipers in a sample of 18 mannequin heads. Soft tissue landmark location was performed digitally in the corresponding software followed by 12 linear measurements (horizontal and vertical) at various regions of the face repeated in two separate sessions. The comparisons between the sessions were insignificant, indicating high precision, while statistically significant differences were found in the distances measured with the three methods; however, these were at the submillimeter level, with the effect sizes being uniformly small (±0.1) and within clinical acceptability. Even though these 3D imaging capture systems produce accurate and repeatable results, the bulk and costs of such specialized equipment are prohibitive for 3D soft tissue imaging in everyday orthodontic clinical practice [6].

The popularity of mobile phones and the development of several applications offer new perspectives in 3D face scanning, making it possible within seconds with affordable costs. Image acquisition using mobile smartphones can be performed in any environment, even by the patients themselves, thus eliminating some of the constraints inherent in other methods, including that professional installation and calibration of the equipment as well as doctor presence for image acquisition are mandatory [16]. Amornvit and Sanohkan 2019 [17] compared measurements performed in all three planes of space (x,y,z) on images captured with various scanners, including two portable scanners (EinScan Pro and EinScan Pro 2X Plus manufactured by Shining 3D Tech. Co., Ltd., Hangzhou, China) and their software, an iPhone X using the Bellus3D Face Application (Bellus3D, version 1.6.2, Bellus3D, Inc., Campbell, CA, USA), Planmeca ProMax 3D Mid (PM) CBCT imaging (Planmeca USA, Inc., Hoffman Estates, IL, USA), and, finally, the direct measurements with calipers served as the controls. Landmarks were marked on a face model that was digitally designed to resemble a human face and printed of polylactic acid. EinScan Pro 2X Plus showed the highest accuracy followed by EinScan Pro with an iPhone X using the Bellus3D Face Application. CBCT showed the lowest accuracy regarding all the methods, but EinScan Pro 2X Plus had problems with measuring depth.

In another study, 40 young adult face scans were obtained using the 3dMDtrio Stereophotogrammetry System (3dMD, Atlanta, GA, USA) as a reference control over an iPhone Xs that used either the Bellus3D Face Application (version 1.6.11; Bellus3D Inc., Campbell, CA, USA) or Capture (version 1.2.5; Standard Cyborg Inc., San Francisco, CA, USA) as the test methods [18]. The images were exported as surfaces that were then aligned and superimposed for comparisons by applying color-mapping to depict surface-to-surface deviations between the methods. The Bellus3D application versus 3dMD matched by 80.0 ± 5.9% and 56.6 ± 7.7% when the thresholds of discrepancy were set at 1 mm and 0.5 mm, respectively, while these values for the Capture application versus 3dMD were 81.4 ± 9.6% and 56.5 ± 11.6% for each respective threshold [18]. Even though the smartphone applications showed promising results, Bellus3D and Capture required an average of 20.3 s and 40.3 s scan time compared to the 3dMD system, which completed the scan in 1.5 milliseconds. Additionally, the processing of the 3D model was completed in less than half the time of the 3dMD system (20.6 s) compared to the two smartphone applications (55.5 s for Bellus3D and 53.5 s for Capture). As a result, the bulk and costs of sophisticated 3D stereophotogrammetry systems are counterbalanced by their accuracy and speed compared to smartphone applications [18].

Given the existing literature, there is limited evidence regarding the accuracy of the Bellus3D Face Application (version 1.6.11; Bellus3D Inc., Campbell, CA, USA) on a smartphone versus direct anthropometric measurements. In addition, there is heterogeneity amongst the existing studies regarding the method of landmark localization and digitization, with some studies performing pre-marking of the landmarks on the model’s face and others performing direct digitization using relevant software. The aim of the present study was to evaluate the accuracy and reproducibility of the measurements obtained using the Bellus3D Face Application on a mobile smartphone by comparing them to direct measurements on pre-marked and blank face scans.

## 2. Materials and Methods

Twenty-five healthy young adults were included in this prospective cross-sectional study (six males and nineteen females) with an age range of 20–30 years. Participants were undergraduate and postgraduate students at the University of Geneva during the academic year 2019–2020 who volunteered to participate in the present study, and the sample size was mainly dictated by the number of individuals who agreed to volunteer. No specific skeletal or facial requirements were essential, with the only exclusion criterion being the presence of significant facial hair interfering with the placement and visualization of landmarks [3,19,20]. For all image acquisitions, an iPhone XR with the Bellus3D FaceApp face scanning application, which is an iOS application for smartphones, was used. This application provides the user with an oval frame on the screen to fit the contours of the face in a frontal view and then instructs the subject to perform rotational movement of the head to the right and left maximum profile in order to carry out a detailed three-dimensional acquisition of the face. This is achieved using hundreds of thousands of projected infrared dots to generate highly accurate facial models, which can then be exported in various formats [21]. To standardize the acquisitions, a smartphone stabilizer (DJI Osmo Mobile, Nanshan, China) was used as well as a headband to keep hair away from the subjects’ faces. All scans were obtained under similar scanning conditions in a room with sufficient and ambient light. Ethics approval was obtained from the University of Geneva commission for ethical research (CUREG-MM-2022-04-65).

### 2.1. Facial Measurements

**Figure 1:**
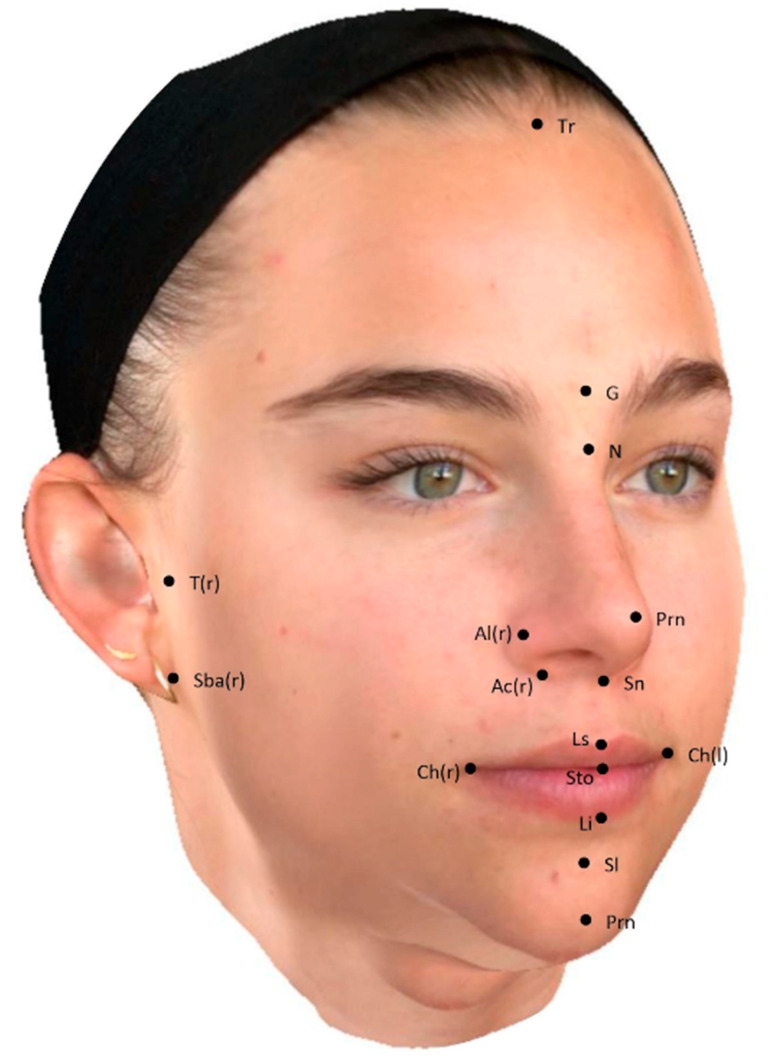
Facial landmarks used. Those shown above are: Ac(r) = right alar crest; Al(r) = right alar; Ch(l) = left cheilion; Ch(r) = right cheilion; G = glabella; Li = labrale inferius; Ls = labrale superius; N = nasion; Prn = pronasale; Sba(r) = right subaural; Sl = sublabiale; Sn = subnasale; Sto = stomion; T(r) = right tragion; Tr = trichion.

The following 20 landmarks were defined as presented in Figure 1 [3,22,23]:

- Midfacial landmarks: Tr (trichion), G (glabella), N (nasion), Prn (pronasale), Sn (subnasale), Ls (labrale superius), Sto (stomion), Li (labrale inferius), Sl (sublabiale), and Pg (pogonion).
- Paired bilateral landmarks: T-l and T-r (right and left tragion), Sba-r and Sba-l (right and left subaural), Al-r and Al-l (right and left alar), Ac-r and Ac-l (right and left alar crest), and Ch-r and Ch-l (right and left cheilion).

An initial three-dimensional acquisition of the face (blank) was carried out with the patient looking straight for 2 s, then turning the head to the right and then back to the center (4 s), and then turning to the left and then back to the center (4 s) following the vocal instructions and the rhythm provided by the application itself and the operator. Then, using an eyeliner pencil, 16 of the landmarks were marked on the subjects’ faces, while Tr, Sto, Ch-r, and Ch-l were not marked but determined anatomically by the same operator following the same conditions and placement sequence for all participants. After marking the face landmarks, a second 3D acquisition was undertaken, followed by measuring the linear distances of interest in the three planes of space using a conventional caliper as follows:

- Vertical measurements: Tr–G, Tr–N, N–Sn, Prn–Sn, Sn–Pg;
- Transversal measures: Alr–All, Chr–Chl;
- Sagittal measurements: Sbar–Chr, Sbal–Chl, Acr–Prn, Acl–Prn.

Once these data from the 3D face scans were collected, the distance measurements were recalculated in .stl format images exported to a personal computer, by a single operator, using VAM software version 6.10 (Vectra, Canfield Scientific Inc., Fairfield, NJ, USA). The distances were digitally measured on both 3D acquisitions of each subject (blank and with the marked landmarks).

To determine the precision and reproducibility of the operator, the points were placed again on ten of the blank face acquisitions and the difference between the first and second values obtained were calculated. The distances measured by the software on blank and marked faces were compared to the distances obtained manually.

### 2.2. Statistical Analysis

Descriptive statistics such as mean and standard deviation (SD) were calculated for each measurement, and Student’s *t*-tests for paired data were used to test the differences between the methods (*p* value threshold < 0.05). The intra-class correlation coefficient was used to check the reproducibility of the different methods of measurement. Dahlberg’s formula was used to assess random error of the placement of points and manual measurements and the placement of virtual points on blank faces by the operator. *T*-tests were used to assess systematic error. All analyses were performed with SPSS v.26 IBM.

## 3. Results

The random error, using Dahlberg’s formula, was found to be 1.0 mm and 0.4 mm for the manual point placement and measurements and virtual point placement on blank faces by the operator, respectively. The two methods used (the direct method and acquisition on faces with landmarks) demonstrate relatively similar reliability (ICC > 0.8, Table 1, Table 2 and Table 3).

- **Differences between virtual measurements on blank faces and direct manual measurements.**

The paired *t*-test showed that the difference between several measurements was less than 1 mm, which was statistically and clinically insignificant; however, significant differences (*p* < 0.05) between the two methods were found for the distances pronasale–subnasale (Prn–Sn), subnasale–pogonion (Sn–Pg), cheilion right–cheilion left (Chr–Chl), pronasale–alar crest right (Prn–Acr), and pronasale–alar crest left (Prn–Acl), all of which include landmarks related to the nose and mouth (Table 1).

- **Differences between virtual measurements on faces with landmarks and direct manual measurements.**

The paired *t*-test showed several statistically significant differences, but not all of them were of clinical significance. The differences that exceeded 1 mm were nasion–subnasale (N–Sn), pronasale–subnasale (Prn–Sn), pronasale–alar crest right (Prn–Acr), and pronasale–alar crest left (Prn–Acl), all of which included landmarks related to the nose (Table 2).

- **Differences between measurements on blank faces and faces with landmarks both performed virtually.**

The paired *t*-test showed several statistically significant differences, but not all of them were of clinical significance. The differences that exceeded 1 mm were pronasale–subnasale (Prn–Sn) and subnasale–pogonion (Sn–Pg), with both of them related to the landmarks of the nose (Table 3).

**Figure 2:**
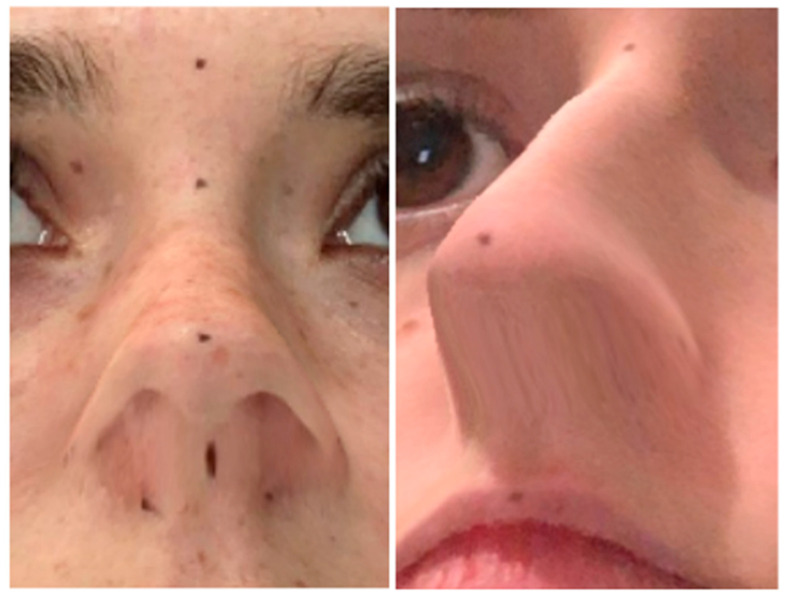
Deviations at the base of the nose.

Regardless of the method used, there was a systematic error for various values that included the nose and mouth (*p* < 0.05) (Figure 2). The measurements demonstrating the most significant differences between the methods were those that included the tip of the nose (Prn–Sn, Prn–Acr, and Prn–Acl), with the averages ranging from −4.4 to 3.3 mm, while the distances ranged from 15.8 to 26.0 mm (*p* < 0.05 and ICC < 0.8).

Contrary to this, the measurements of the distances that estimate face “depth” (Sbar–Chr and Sbal–Chl) showed the greatest consistency irrespective of the tested method (*p* > 0.05 and ICC > 0.8).

## 4. Discussion

In the present study, comparisons were performed regarding linear measurements of the face between two virtual acquisition methods (blank faces and faces marked with landmarks) using the Bellus 3D FaceApp and a smartphone versus direct conventional measurements determined manually, with the latter considered to be the current gold standard. The applicability and accuracy of the system were assessed on several variables used in facial anthropometry [3,9,19]. The linear distances ranged from 15 to 110 mm and represent several measurements that could be useful in clinical practice [3]. The results showed that the Bellus 3D FaceApp has good reliability and reproducibility for the areas of the face that do not include the nose and the corners of the mouth. Marking the landmarks on the face made it possible to quantify the deformation due to the Bellus3D FaceApp by comparing the measurements from the virtual acquisitions on blank faces and faces with landmarks. Since manual measurements and point placement on blank faces are considered to be valid (random error = 1.0 mm and 0.4 mm, respectively), the most notable differences were regarding those that involved the mouth and the tip of the nose. This could be attributed to the deformation of this anatomical area by the software and image acquisition method and must be considered when planning or simulating orthodontic, surgical, or dental interventions that might affect the projection of these areas relative to the face and especially the lips.

The discrepancies between the various measurements can be due to the intrinsic error related to the application’s limitations, uncoordinated movement, incorrect head position [16], or involuntary facial movements that can reach 1.5 mm in the critical areas of the soft tissues, such as those of the eyes, mouth, and nose [6,24]. Additionally, involuntary facial expressions may also play a role in these discrepancies, as reported in the literature [3,7]. Regarding 3D acquisition on blank faces, the measurements of nose width (Alr–All) were reproducible and reliable. These findings are in agreement with a systematic review that compared the accuracy of face scans obtained using mobile smartphones with professional 3D facial scanning equipment [25]. The standardized mean difference in the global meta-analysis between the mobile and professional scanners was 3.96 mm (95% CI 2.81–5.10 mm; z = 6.78; *p* < 0.001), a finding that is both statistically and clinically important, indicating the inferior performance of smartphones over more sophisticated equipment. Additionally, the performance was much better on artificial mannequins than living participants, which raises caution regarding the clinical applicability, where practitioners have to treat real patients, as well as in the interpretation of the results from studies that use different samples as models for performing the comparisons across different methods for image acquisition [25].

Similarly, a network meta-analysis investigating the accuracy of various face-scanning technologies [static or portable stereophotogrammetry (s-SP or p-SP), structured light (SL), laser scanner (LS), infrared structured light (ISL), and LED-structured light (LSL)] versus direct anthropometry showed that the mean differences in the distances between the considered landmarks were 1.10 to −1.74 mm [26]. The outcomes included in the quantitative comparisons in the meta-analysis were the distances En–En [right and left Endocantion (En) medial corner of the eye points], Ex–Ex [right and left Exocantion (Ex) lateral corner of the eye points], Al–Al [right and left alar points located at the wing of the nose], Ch–Ch [right and left Chelion (Ch) points located at the corners of the mouth where the upper and lower lips join], N–Sn [nasion and subnasale, both single points located at the root of the nose and the midpoint between the base of the columella and the upper lip, respectively], T–Pg [Tragus (t) triangular protrusion near the auricle, and Pogonion (Pg) the most anterior point of the “tip” of the jaw]. It is obvious that most of these points and outcomes were common to the ones used in the present report. The pooled differences between the virtual measurements on faces with landmarks and the direct manual measurements were generally less than 1 mm, being clinically insignificant; however, they were lower regarding the differences in the present study, especially for the distances defined by the landmarks of the nose and lips. This can be attributed to the greater samples and inclusion of various scanning modalities and technologies used for the calculation of pooled effects in the network meta-analysis [26].

In another study, the Bellus3D app system on an Apple iPhone 11 Pro [IOS 14.0.1] with a “TrueDepth” camera system was compared to the 3dMDface system by registering the two facial surface scans on the forehead and bridge of the nose using the best-fit algorithms [27]. The root mean square (RMS) differences calculated by the ‘deviation analysis’ function in the Geomagic Control X software (3D systems, Rock Hill, SC, USA), using surface-to-surface and point-to-point measures between the two registered surfaces, accounted for 0.86 ± 0.31 mm. For 97% of the used landmarks, the error was within 2 mm, indicating the clinical accuracy and reliability of the exact conditions and systems used to capture the relevant face scans [27]. A similar methodology of surface superimpositions compared the overlays obtained using either Zirkonzahn’s Face Hunter scanner or the Bellus 3D Dental Pro app on an iPhone X, while the linear distances were also measured digitally on the scans and compared to the manual measurements [28]. It was found that the cheeks had the highest reproducible range of overlap (60%), followed by the chin and the tip of the nose [28]. Contrary to the previous reports, the Bellus3D Dental Pro app versus soft tissue shells segmented from CBCTs showed statistically and clinically significant differences ˃3 mm in some areas of the face, which is a clinically significant discrepancy not to be overlooked when treatment planning is based on such face scans [29]. The surface superimpositions indicated that the lowest total deviation (≤10%) was at the central regions of the nose (bridge and tip), the central regions above and below the upper and lower lips, as well as the areas of the zygomatic bones and cheeks. Higher deviations were noted for the lateral areas of the nose and the orbital regions, accounting for more than 30% and 60% of these deviations, respectively [29]. The authors report that the current smartphone technology has limited applicability for clinical use, especially in those areas of the face that require increased precision [29]. Nevertheless, the low deviations found at the tip of the nose contradict the results of the present report. This disagreement could be attributed to the different methods used as, in the present report, we did not apply surface superimpositions but linear measurements performed either digitally or with calipers.

The most repeatable distances found in the present report involved facial depth and vertical measurements that did not include the nose. For example, the values of Sbar–Chr and Sbal–Chl (the base of the ear to the corner of the lip bilaterally) are important in the quantitative analysis of facial features [3]. In the present study, these did not show significant differences irrespective of the method used and despite the difficulties in digitization and the possible sources of distortion. In general, the most striking variations were consistent for specific areas irrespective of the subject’s facial morphology or sex. Additionally, placing landmarks before image acquisition does not significantly reduce errors compared to acquisitions on blank faces [3,6,13,20]. Thus, the advantages of this method remain in its ease of use, the absence of the background of the environment, and the accessibility and compatibility with smartphones. Based on the results of the present study, virtual point placement on a blank face was more accurate than manual point placement and measurement, which makes smartphone image acquisition a reference in everyday clinical practice. However, the application works only after face detection, and the determination of its accuracy cannot be performed against a geometric object of known dimensions [3,4,16].

## 5. Generalizability and Limitations

The study sample was relatively small and was composed predominantly by young females; however, this was dictated also by the number of individuals who volunteered to participate in the study. In addition, individuals with significant facial hair were excluded from the study sample due to the difficulties in locating and marking the facial landmark points. This means that the results of the present study are applicable to young females and individuals without facial hair and can also be used as baseline information for the sample size calculations of future studies.

## 6. Conclusions

The use of the Bellus3D FaceApp is precise and reproducible for certain areas of the face; however, digital reconstruction errors exist. In general, the noted differences between the virtual and manual measurements were less than 1 mm, rendering the clinical differences insignificant. Discrepancies of more than 1mm were consistently noted for specific measurements that included as landmarks the tip and the base of the nose. Further investigations are required to determine other sources of error and for other smartphone-based applications released for 3D face image acquisitions.

## Figures and Tables

**Table 1:** Descriptive statistics, mean and standard deviation (SD), and the differences between virtual measurements on blank faces and direct manual measurements.

	Virtual Measurements on Blank Faces (mm)		Manual Measurements (mm)		Difference Between Methods (mm)			
Distances	Mean	SD	Mean	SD	Mean	SD	*p*	ICC
Tr–G	62.2	8.6	61.9	8.4	0.3	1.0	0.21	0.99
Tr–N	75.2	8.1	74.8	8.6	0.5	1.1	0.06	0.99
N–Sn	50.2	3.7	49.7	3.7	0.5	1.6	0.16	0.90
Prn–Sn	15.8	2.9	20.2	2.5	−4.4	2.3	0.00	0.01
Sn–Pg	53.6	5.2	52.3	4.8	1.2	2.0	0.00	0.90
Alr–All	32.8	3.4	32.7	3.7	0.2	1.0	0.47	0.96
Chr–Chl	44.7	4.0	45.9	3.8	−1.1	1.2	0.00	0.91
Prn–Acr	22.3	2.4	26.0	2.4	−3.7	1.4	0.00	0.14
Prn–Acl	22.1	2.4	25.8	2.5	−3.8	1.4	0.00	0.15
Sbar–Chr	92.1	6.9	91.9	6.4	0.2	2.2	0.70	0.95
Sbal–Chl	92.0	6.7	92.1	6.4	−0.2	2.9	0.78	0.91

Tr–G: trichion–glabella; Tr–N: trichion–nasion; N–Sn: nasion–subnasale; Prn–Sn: pronasale–subnasale; Sn–Pg: subnasale–pogonion; Alr–All: alar right–alar left; Chr–Chl: cheilion right–cheilion left; Prn–Acr: pronasale–alar crest right; Prn–Acl: pronasale–alar crest left; Sbar–Chr: subaural right–cheilion right; Sbal–Chl: subaural left–cheilion left.

**Table 2:** Descriptive statistics, mean and standard deviation (SD), and the differences between virtual measurements on faces with landmarks and direct manual measurements.

	Virtual Measurements on Faces with Landmarks (mm)		Manual Measurements (mm)		Difference Between Methods (mm)			
Distances	Mean	SD	Mean	SD	Mean	SD	*p*	ICC
Tr–G	61.6	8.2	61.9	8.4	−0.3	0.7	0.09	0.99
Tr–N	75.1	8.3	74.8	8.6	0.3	0.6	0.01	0.99
N–Sn	51.0	3.7	49.7	3.7	1.2	1.0	0.00	0.91
Prn–Sn	19.1	2.0	20.2	2.5	−1.1	0.9	0.00	0.81
Sn–Pg	51.7	4.8	52.3	4.8	−0.6	1.0	0.01	0.97
Alr–All	32.3	3.5	32.7	3.7	−0.4	0.7	0.01	0.97
Chr–Chl	45.4	3.9	45.9	3.8	−0.5	1.1	0.03	0.96
Prn–Acr	23.1	2.1	26.0	2.4	−2.9	1.3	0.00	0.31
Prn–Acl	22.8	2.1	25.8	2.5	−3.0	1.5	0.00	0.27
Sbar–Chr	91.9	7.2	91.9	6.4	0.0	2.3	0.98	0.94
Sbal–Chl	91.6	6.3	92.1	6.4	−0.5	3.0	0.41	0.89

Tr–G: trichion–glabella; Tr–N: trichion–nasion; N–Sn: nasion–subnasale; Prn–Sn: pronasale–subnasale; Sn–Pg: subnasale–pogonion; Alr–All: alar right–alar left; Chr–Chl: cheilion right–cheilion left; Prn–Acr: pronasale–alar crest right; Prn–Acl: pronasale–alar crest left; Sbar–Chr: subaural right–cheilion right; Sbal–Chl: subaural left–cheilion left.

**Table 3:** Descriptive statistics, mean and standard deviation (SD), and the differences between measurements on blank faces and faces with landmarks both performed virtually.

	Virtual Measurements on Blank Faces (mm)		Virtual Measurements on Faces with Landmarks (mm)		Difference Between Methods (mm)			
Distances	Mean	SD	Mean	SD	Mean	SD	*p*	ICC
Tr–G	62.2	8.6	61.6	8.2	−0.5	0.9	0.01	0.99
Tr–N	75.2	8.1	75.1	8.3	−0.1	1.0	0.46	0.99
N–Sn	50.2	3.7	51.0	3.7	0.8	1.2	0.00	0.93
Prn–Sn	15.8	2.9	19.1	2.0	3.3	2.0	0.00	0.17
Sn–Pg	53.6	5.2	51.7	4.8	−1.8	1.9	0.00	0.87
Alr–All	32.8	3.4	32.3	3.5	−0.6	0.8	0.00	0.96
Chr–Chl	44.7	4.0	45.4	3.9	0.7	0.9	0.00	0.96
Prn–Acr	22.3	2.4	23.1	2.1	0.8	1.3	0.00	0.77
Prn–Acl	22.1	2.4	22.8	2.1	0.8	1.4	0.01	0.75
Sbar–Chr	92.1	6.9	91.9	7.2	−0.2	1.8	0.63	0.97
Sbal–Chl	92.0	6.7	91.6	6.3	−0.3	2.1	0.42	0.95

Tr–G: trichion–glabella; Tr–N: trichion–nasion; N–Sn: nasion–subnasale; Prn–Sn: pronasale–subnasale; Sn–Pg: subnasale–pogonion; Alr–All: alar right–alar left; Chr–Chl: cheilion right–cheilion left; Prn–Acr: pronasale–alar crest right; Prn–Acl: pronasale–alar crest left; Sbar–Chr: subaural right–cheilion right; Sbal–Chl: subaural left–cheilion left.

## Data Availability

Data are available on reasonable request.
